# How Much Does It Cost to Improve Access to Voluntary Medical Male Circumcision among High-Risk, Low-Income Communities in Uganda?

**DOI:** 10.1371/journal.pone.0119484

**Published:** 2015-03-16

**Authors:** Bruce Larson, Allan Tindikahwa, George Mwidu, Hannah Kibuuka, Fred Magala

**Affiliations:** 1 Center for Global Health and Development, Boston University, Boston, Massachusetts, 02118, United States of America; 2 Department of International Health, Boston University School of Public Health, Boston, Massachusetts, 02118, United States of America; 3 Makerere University Walter Reed Project, Kampala, 256, Uganda; Örebro University, SWEDEN

## Abstract

**Background:**

The Ugandan Ministry of Health has endorsed voluntary medical male circumcision as an HIV prevention strategy and has set ambitious goals (e.g., 4.2 million circumcisions by 2015). Innovative strategies to improve access for hard to reach, high risk, and poor populations are essential for reaching such goals. In 2009, the Makerere University Walter Reed Project began the first facility-based VMMC program in Uganda in a non-research setting. In addition, a mobile clinic began providing VMMC services to more remote, rural locations in 2011. The primary objective of this study was to estimate the average cost of performing VMMCs in the mobile clinic compared to those performed in health facilities (fixed sites). The difference between such costs is the cost of improving access to VMMC.

**Methods:**

A micro-costing approach was used to estimate costs from the service provider’s perspective of a circumcision. Supply chain and higher-level program support costs are not included.

**Results:**

The average cost (US$2012) of resources used per circumcision was $61 in the mobile program ($72 for more remote locations) compared to $34 at the fixed site. Costs for community mobilization, HIV testing, the initial medical exam, and staff for performing VMMC operations were similar for both programs. The cost of disposable surgical kits, the additional upfront cost for the mobile clinic, and additional costs for staff drive the differences in costs between the two programs. Cost estimates are relatively insensitive to patient flow over time.

**Conclusion:**

The MUWRP VMMC program improves access for hard to reach, relatively poor, and high-risk rural populations for a cost of $27-$38 per VMMC. Costs to patients to access services are almost certainly less in the mobile program, by reducing out-of-pocket travel expenses and lost time and associated income, all of which have been shown to be barriers for accessing treatment.

## Introduction

Voluntary medical male circumcision (VMMC) significantly reduces the risks of acquiring HIV in men.[1–6] In Uganda, where HIV prevalence among men 15–59 is estimated at 6.1%, approximately 26% of men aged 15–49 are circumcised, and 46% of uncircumcised men say they would like to complete the operation.[7] The Ministry of Health (MOH) formally endorsed VMMC as an effective HIV prevention strategy in 2010 and set a goal of circumcising 4.2 million Ugandans by 2015.[8] The Uganda AIDS Commission's (UAC) annual review of the National HIV and AIDS Strategic Plan (2011–12) reported that 380,000 men received VMMC by March 2012.

In 2009, the Makerere University Walter Reed Project (MUWRP), which is a non-profit partnership between Makerere University in Uganda and the U.S. Military HIV Research Program (centered at Walter Reed Army Institute for Research in the United States), began the first VMMC program in Uganda in a non-research setting. Between February and September 2009, MUWRP conducted a pilot program in two sub-counties in Kayunga district. Following the successful implementation of the pilot program, the MUWRP expanded VMMC services to men living in or near Kayunga and Mukono Districts (Region Central 2). In addition to providing VMMC operations at health facilities, a mobile VMMC clinic began providing VMMC services to more remote, rural locations (compared to the three health facilities providing VMMC services) in 2011.

Although several analyses have generally evaluated the cost-effectiveness of male circumcision in different settings[2, 3, 5, 9–14], little information exists to compare the costs of facility-based and mobile VMMC service delivery. Fixed sites tend to be located in urban or peri-urban locations. The mobile program in Uganda was established specifically to improve access to VMMC services more remote, high-risk, and low-income populations in roughly the same overall region. As summarized in the overview of the PLOS Collection on Voluntary Medical Male Circumcision for HIV Prevention, and additional articles addressing demand creation, basic availability is a key predictor of VMMC uptake.[15] Using information extracted from MUWRP program records and in collaboration with program staff, the primary objective of this study was to estimate the average costs of performing VMMCs in the mobile clinic and to compare these costs to the average cost of VMMCs performed in health facilities (fixed sites). The difference in these costs, in effect, shows the cost of reducing barriers to access to VMMC and improving targeting, both of which have been identified as key issues for achieving ambitious national VMMC goals.[15–18] Information developed as part of this study will also inform policy makers, program managers, and funders about variation in the costs of VMMC delivery, and the main drivers of those costs in Uganda, between facility-based and mobile clinic service delivery.

## Methods

### Study setting

The MUWRP VMMC program supports activities in Kayunga, Buvuma and Mukono Districts of Uganda (Region Central 2). Facility-based VMMC is currently provided at Kayunga District Hospital and at Kojja Health Center IV and Mukono Health Center IV, with VMMC operations performed in newly renovated “minor” theaters with 2 surgical beds in each facility dedicated to VMMC operations. The mobile clinic also provides VMMC services in Kayunga and Mukono Districts, focusing on remote villages where men do not have local access to VMMC services, including remote fishing villages near Lake Kyoga in Kayunga District with high rates of HIV. Dorsal slit and forceps-guided are the primary techniques of circumcision used in the program.

Adverse events requiring extra medical care following VMMC performed by this program are rare. For example, the MUWRP conducted a pilot program in two sub-counties in Kayunga district. Clinical outcome data on 316 de-identified clients were reviewed retrospectively at one, four and fifty two weeks post-surgery (Protocol RV 277).[19] By 4 weeks after the procedure, there were no reports of difficulty passing urine, pain in the penis, complaints, significant findings, or any signs of infection.

This VMMC program, both at three fixed sites and the mobile unit, utilizes staff (clinical officers and nurses) trained by the Ugandan Ministry of Health (MOH) for performing operations and monitoring clients. The same staff members work in the fixed sites and in the mobile program, and receive the same training. The MUWRP receives funding from U.S. President’s Emergency Plan for AIDS Relief (PEPFAR) for staffing, supplies and related activities (e.g. renovating minor surgical theaters at sites).

The mobile VMMC clinic is a truck with a self-contained surgical unit and two surgical beds mounted on the frame. A generator provides electricity for the unit (including air conditioning). The mobile program began providing services in March of 2011. The mobile clinic is “based” at a fixed site (Kayunga District Hospital), and the MUWRP program manages the overall VMMC program (the fixed sites and mobile clinic).

### Description of program

For both fixed and mobile sites, the overall VMMC process begins with community mobilization and recruitment activities to generate demand for the service (called Step 0). Once a recruit presents at a mobile or fixed site, the patient then proceeds through three main steps: (1) VMMC counseling and HIV testing; (2) completing a medical exam to confirm eligibility for the procedure that day; and (3) completing the surgical procedure (with post-operation follow up). Step 1 also includes obtaining consent from the patient for the procedure. At each of these stages, various supplies, equipment, and staff are used. The costing analysis presented here organizes the costs for each stage of the VMMC process.

While called the “mobile” program, the mobile unit is driven to a location where it remains for several days (typically day 1 is driving to a site, operations are then performed for the next 12 days (with no days off), and then the vehicle returns to the home base near Kayunga District Hospital at the end of the period. For locations that are not too far away from the home base, the staff travel each day to the site (in a separate vehicle) for work and then return home in the evening. This type of location is called “staff returning”. For locations that are more distant and/or with more difficult roads, staff members remain with the mobile unit throughout the period and find accommodations and meals in the local community. The program calls this a “camping” location in the sense that staff members are lodged in nearby towns rather than returning to their homes each evening. Costs for each type of mobile program, staff returning and staff camping, along with costs for a fixed site, are included in this analysis.

At the fixed site, waste is managed according to standard procedures at the site. A sanitary officer is included in staff costs for managing this waste, and other services such as incineration are included as part of the facility management and administration costs. For the mobile program, waste is collected during the day and the returned with the staff (the staff returning model). With a camping location, staff members are housed in locations with hotels, which are also in communities with government health facilities with incinerators. In this case, the daily waste (typical 2 moderate sized plastic bags) is delivered to such facilities. The program pays no fee for this service, and no additional costs for this service is included because of the small quantities involved.

### Costing Perspective and Data collection

This program evaluation focuses on the costs of VMMC performed in the mobile program and comparing to costs of procedures performed in a typical fixed site. All costing information is based on routine program implementation records. No human subjects data were used in this analysis.

All costs above the service-delivery level, such as MUWRP program management, costs of training staff, supply chain, and so on, are excluded from this analysis. This approach is similar to previous analyses focusing on direct costs of VMMC.[20] These higher-level costs are largely fixed costs for the overall VMMC program, so they are less relevant for comparing costs between VMMCs performed in fixed sites and in the mobile program. Experience from another program in Swaziland suggested that supply chain costs alone could add significantly to total program costs.[21]

A micro-costing approach, from the perspective of the program implementer, was used to document and account for resources used for completing VMMC procedures through both service delivery models.[22] All unit costs for resources used (inputs) were based on costs to the MUWRP program (e.g. program invoices), standard MUWRP staff salary scales, and local retail prices (e.g. minor items procured locally in small amounts). All resources typically used at fixed and mobile sites for VMMC services are included in costs. All costs are reported in 2012 Ugandan Shillings (UGX). Cost for other years, such as supplies purchased in 2011 but not used until 2012, were inflated to 2012 using consumer price inflation from the Central Bank of Uganda (www.bou.or.ug/bou/rates_statistics/statistics.html). Items purchased in other currencies (USD) were converted to UGX based on the annual average exchange rate for the purchase year (Oanda.com interbank rate). Equipment costs were converted to a monthly equivalent cost based on expected working life and a 3% real annual discount rate. All costs are expressed as cost per circumcision.

Although the VMMC procedure is provided free-of-charge in both the fixed and mobile clinics, patients incur other costs, such as the opportunity cost of their time to travel to a clinic for a procedure and recovery as well as any out-of-pocket expenses. While these costs are excluded from this analysis, such costs will influence whether a patient presents to a facility for a procedure as well as at which location.

### Assumptions and calculations provided in supplemental digital content

All information, assumptions, and calculations developed and used for this analysis are presented in two Excel files provided as supplemental digital content (S1 Costs Mobile; S1 Costs Fixed). Each Excel file contains several worksheets, with information linked across sheets as needed. Each worksheet within each file contains one or more tables needed for the analysis of that stage (or portion of a stage), and all calculations are included in these worksheets so that readers can evaluate easily how results would change with different assumptions and program conditions.

For the mobile program, the worksheet labeled “preliminary information” contains a Table 1 summarizing basic assumptions and program information used throughout the analysis. A “scale parameter” is included in Table 1 to allow sensitivity analyses of program scale to be easily estimated (1 = scale for 2012). With 30 clients per day at capacity for the mobile program (when using the 2 surgical beds in the mobile unit), scale is driven by the number of days the mobile unit is in operation during the year. For ease of reviewing results of various sensitivity analyses, a summary table showing costs per VMMC disaggregated by steps in the VMMC process is also provided in the preliminary information sheet. The next worksheet (0. Community Mobilization) shows the typical costs the program incurs for such activities.

**Table 1:** Preliminary information for mobile program and fixed sites.

**A. Mobile Program**	
Costing year	2012
Total number of VMMCs performed during year in mobile unit	3605
Today days mobile unit used during year (when circumcisions performed)	120
Days mobile unit used on average per month	10
Average working days unit stays at one location (same fixed and camping)	12
Number of locations with staff camping	4
Days staff camping with mobile unit (circumcision days)	48
Days mobile unit at site but staff return each day (circumcision days)	72
Number of locations with staff returning daily	6
Total locations annually	10
Clients per day completing VMMC	30
Proportion of recruited clients who do not complete procedure	0.01
Clients per day recruited for VMMC	30
Discount rate used	0.03
Average distance site from mobile unit base (Kayunga) if location with staff returning daily (one way, kilometers)	45
Average distance site from mobile unit base (Kayunga) if location with staff camping (one way, kilometers)	120
Total distance mobile unit moves if location with staff returning daily (per location)	90
Total distance additional staff vehicle travels with staff returning daily (per location)*	1260
Total distance mobile unit travels if location with staff camping (per location)	240
Total distance additional staff vehicle moves with staff camping (per location)	264
Average cost of fuel per liter (UGX)	2800
* Assumes 2 extra travel days per staff returning site for the staff vehicle to assist with set up and take down.	
	
**B. Fixed Sites**	
Costing year	2012
VMMCs completed annually (2012 fixed site)	3298
VMMCs completed monthly	274.8
Days providing VMMC services annually	247
Clients per day completing VMMC	13
Proportion of recruited clients who do not complete procedure	0.01
Total individuals recruited for MC (number rounded)	13
Discount rate used	0.03
	
** Other Information**	****
Exchange rates (Oanda.com annual average)	
UGX/$ 2012	2469
UGX/$ 2010	2153
UGX/$ 2011	2493
ZAR/$ (South Africa Rand) 2011	7.23
	
Inflation index numbers	
Year	
2009	139.6
2010	145.2
2011	172.3
2012	196.4
Source: Uganda Bureau of Statistics	

The worksheet “1. VMMC Ed and HCT” organizes costs associated with VMMC education, counseling and consenting and HIV counseling and testing. These activities take place in a tent set up near the mobile unit, and the VMMC ED and HCT sheet first organizes costs for equipment used directly for this stage (e.g., tent, chairs, tables, etc.). Once the costs per month for such equipment are calculated, the costs per patient for this step (equipment, staff/counselors, and materials) are estimated (see Table Step 1. VMMC Education and HCT—Total). Step 2 is the medical exam, which is completed by a clinical officer who provides the medical exam and then also performs the operation on the same patient (so salary costs for the clinical officer for all activities are included as part of Step 3). The exam is also completed in a tent set up near the mobile unit, and some additional basic equipment is used during the exam (HemoCue, etc.).

Step 3 is the actual procedure, and three separate worksheets are used to organize costs for this step and for any other costs not included in the previous steps. The worksheet “S3. Equipment and Other” covers equipment costs, including the costs for purchasing and importing the mobile unit from South Africa, and various other supplies and services used in the mobile program (e.g., insurance, generator and fuel, additional vehicle for moving staff, and so on). (The cost of purchasing and importing the mobile unit from South Africa, including freight charges, was about USD $250,000 in 2011). Because locations with staff returning are closer than camping locations (an estimated 45 kilometers compared to 120 kilometers on average for 2012), the same worksheet “S3. Equipment and Other” estimates costs for a location with staff returning daily and for camping locations separately. The sheet labeled “S3. Staff salaries and costs” first shows standard salaries for various types of program staff, from which standard daily wages are estimated.

The table labeled “S3. Staff Costs per Procedure”, which exclude staff costs from Step 1, then estimates salary costs based on the number of staff used in various capacities (clinical officers assisted by nurses perform the operations along with drivers and sanitary officers providing additional support). Patients are counseled to return after 7 days and one month for follow up visits. At the fixed sites, these services are provided on top of performing circumcisions by the same staff, so the analysis includes such services. For the mobile program, a clinical officer remains at the site for 7 days to complete the initial 7 day follow up visit (salary and per diem based on returning or camping). These costs are included in the analysis. At the 7-day follow up visit, patients receiving services in the mobile program are then referred to their local health facility for the 4-week follow up visit. The cost of providing the 4-week follow up visit is not included in this analysis for the mobile program. The sheet labeled ‘S3. Supplies for VMMC procedure (not included elsewhere)’ includes a list of supplies used during the procedure itself, the most important of which is the disposable surgical sets used in the mobile program.

The final sheet in the Excel file is labeled “Total cost per VMMC”, which reports costs separately for VMMC performed at sites with staff camping and staff returning (in UGX and USD). A separate Excel file shows the analysis for the fixed site and is organized in a similar fashion. The analysis for Steps 0, 1, and 2 are essentially the same for the fixed and mobile programs. For Step 3, the fixed site has only one model of service delivery (as compared to the staff camping or staff returning models for the mobile program). A main difference between the mobile and fixed sites is that the mobile program uses disposal surgical kits, while the fixed sites use reusable surgical supplies (e.g. that are washed and sterilized after each use). Thus, the list of supplies used in Step 3 is substantially longer than the list for the mobile program. Three fixed sites are providing VMMC services as part of the MUWRP. Staffing and resources used are consistent across sites. For this analysis, information on the costs of facility renovation, utilities, and patient flow are based on the Mokono Health Center.

## Results

Table 1 describes program implementation parameters used throughout the analysis. In 2012, the mobile unit performed procedures during 120 days, with 12 working days on average in a location. During the year, 10 locations total were serviced (4 with staff camping and 6 with staff returning). On average, 30 procedures were completed daily (roughly one every 30 minutes during an 8 hour day). For the fixed site, operations were performed on 247 days during 2012, with 13 operations performed on average per day.

Table 1 also highlights the distances traveled by the mobile surgical unit and the additional staff vehicle for locations with staff returning compared to staff camping. For either type of location, the mobile surgical unit makes only one round trip (90 kms round trip with staff returning compared to 240 kms with staff camping at more remote camps). However, for a staff returning location, the additional vehicle for staff travels an additional 1260 kilometers over the stay at each location to deliver staff daily to the site and back home compared to an estimated 264 kms with staff camping (just one round trip with additional minor local travel).

### Cost per VMMC in the mobile program

Based on program information in 2012, we estimate that the cost to complete one VMMC in the mobile program was $60.79 for locations with the staff returning and $72.21 for locations with staff camping (see Table 2). The difference of about $11 is driven by the difference in staff costs ($20.89 camping compared to $8.86 for staff returning) due mainly to the extra costs to house and feed staff local for the camping period. Community mobilization, VMMC education and HIV testing, and performing the medical exam contribute minor amounts to overall costs. Little room exists to reduce VMMC costs from adjustments to these portions of the VMMC process. Costs for Step 3, disaggregated into costs for vehicles, other equipment and other supplies, staff, and materials consumed per procedure, drive overall VMMC costs for the program. Vehicles, other equipment and other supplies, estimated at $18.17 with staff returning locations and $17.63 for staff camping locations, include the mobile unit itself along with multiple related costs (insurance, fuel for the generator, maintenance/servicing agreements), security, and use of the other vehicle transporting staff members. Supplies used during the VMMC procedure are estimated at $27.70, the majority of which is the disposable surgical kit (at $23).

**Table 2:** Estimated cost per VMMC for 2012 program conditions (USD).

**Steps**	**Worksheet name** ^1^	**MOBILE Staff returning**	**Percentage of estimated cost**	**MOBILE Camping**	**Percentage of estimated cost**
Step 0	Community Mobilization	1.91	3%	1.91	3%
Step 1	VMMC Education and HCT	3.41	6%	3.41	5%
Step 2	Exam	0.56	1%	0.56	1%
Step 3	Staff	8.86	15%	20.89	29%
Step 3	Vehicle, Equipment and Other Supplies	18.34	30%	17.74	25%
Step 3	Supplies for VMMC procedure	27.70	46%	27.70	38%
	Estimated cost per VMMC (2012)	60.79	100%	72.21	100%
**Steps**	**Worksheet name** ^**2**^	**Fixed Site**	**Percentage of estimated cost**		
Step 0	Community Mobilization	0.29	1%		
Step 1	VMMC Education and HCT	2.98	9%		
Step 2	Exam	0.31	1%		
Step 3	Staff	12.40	36%		
Step 3	Facility costs	12.02	35%		
Step 3	Equipment and Supplies	6.19	18%		
	Estimated cost per VMMC (2012)	34.20	100%		

^1^ The worksheets are found in the supplemental digital content (S1 Costs Mobile and S1 Costs Fixed).

### Costs per VMMC at fixed sites

We estimate that the cost to complete one VMMC was $34 at the fixed site. As with the mobile program, costs for community mobilization, VMMC education and HIV testing, and the exam contributed minor amounts to costs per VMMC. The cost categories for Step 3 differ somewhat between the mobile and fixed sites, and the categories are lined up in Table 2 to be relatively comparable. For the fixed site, facility costs per VMMC are less than similar costs in the mobile program (the category “equipment and other supplies” includes the cost of the mobile unit), but not substantially less because of the major renovations needed at the fixed sites for the minor theaters used for VMMC operations (around $100,000). Staff costs of $12.40 are between staff cost estimates for the mobile program ($7.97 when staff members return home each evening and $20.02 at camping sites). Supplies used during the VMMC operation for the fixed sites are estimated at $12.90.

## Discussion

Improving access to VMMC through a mobile VMMC program that brings the service to remote, rural, low-income, and high-risk communities is estimated to cost between $27–$38 per VMMC.

The cost of the disposable surgical kit, $23 in 2012, was the single largest component of cost in the mobile program, larger than total equipment costs per procedure or total salary costs of staff. Reductions in the price of these kits (local cost in Uganda including all related costs and taxes if imported) will translate into direct reductions in the costs per procedure. For comparison, supplies used during the VMMC operation for the fixed sites are estimated at only $12.90, mainly because the fixed sites do not use the disposable surgical kits.

Another key difference is patient flow. In the mobile program, on the days when procedures were performed, 30 procedures were completed daily (roughly one every 30 minutes during an 8 hour day with two surgeons). In contrast, 13 operations were performed on average per day. This difference in average procedures per day reflects the fact that fixed sites provides services almost every working day during the year, while days per year are fairly limited for any one location visited by the mobile unit.

These cost estimates are fairly robust to wide ranges of alternative assumptions regarding equipment and patient flow. Raising the discount rate to 10% increases cost per VMMC in the mobile program by about $3. Extending the mobile unit’s working life from 10 years to 15 years reduces cost by about $3. Increasing patient flow in the mobile program from 30 to 40 procedures per day (from 15 to 20 patients per surgeon per day) reduces costs by a minor amount, while cutting patients to only 15 per day total increases costs by a minor amount ($2). In the fixed program, increasing patient flow to 30 patients per day reduces costs by about $7 (to $27 total). If the mobile unit operated 240 days during the year, compared to 120 actual days during 2012, costs per procedure would fall by $6–7.

Even if a mobile unit and all related costs (insurance, maintenance contracts) could be procured for 50% less than the actual costs, costs would only fall by $7/6 for staff returning/camping locations. When demand for VMMCs is expected to be high at a site, the MUWRP VMMC program is able to bring along extra capacity (an extra tent large enough for two surgical beds) to perform circumcisions. This approach essentially allows the program to spread out the upfront costs for the mobile unit across more procedures, recognizing some additional costs associated with the additional test and related surgical equipment (e.g. surgical beds, lamps).

Rather than reducing the cost of access by bringing the service closer to the targeted communities, a program could also consider alternative approaches such as paying the patient to travel to a fixed clinic. For example, in a recent study from Kenya, which randomized men to 4 payment levels if they completed a VMMC at one of the study sites (nothing, $2.50, $8.75, $15.00 in food voucher equivalents), a statistically significant but absolutely small improvement in VMMC uptake after 2 months was observed in the two higher-payment groups compared to the lower two groups (e.g. from about 2% up to about 7–9%).[17]

Evaluating health service delivery requires more information than just costs to the provider. The question that cannot be addressed in this paper is if the estimated difference in average costs between the fixed and mobile settings is reasonable based on additional information that should logically be considered by policy makers. By targeting rural locations with relatively high HIV prevalence, fewer VMMCs would need to be performed to avert one HIV infection. For patients in remote locations, their costs to access services are certainly less in the mobile program compared to fixed sites. If communities in more remote locations also are poorer than communities near fixed sites, the mobile program also improve equity of access to health. Targeting communities that are relatively remote, poor, and with high HIV risks are logical strategies to improve the cost effectiveness of mobile VMMC programs based on cost per infected averted.[15]

## Supporting Information

S1 Costs Mobile (XLSX)

S1 Costs Fixed (XLSX)
